# Age-Associated Changes to Lymph Node Fibroblastic Reticular Cells

**DOI:** 10.3389/fragi.2022.838943

**Published:** 2022-01-25

**Authors:** Tina Kwok, Shannon C. Medovich, Ildefonso A. Silva-Junior, Elise M. Brown, Joel C. Haug, Marliece R. Barrios, Karina A. Morris, Jessica N. Lancaster

**Affiliations:** Department of Immunology, Mayo Clinic, Scottsdale, AZ, United States

**Keywords:** lymph nodes, fibrosis, lymphotoxin, T cell aging, live imaging, naive T cells, two-photon microscopy

## Abstract

The decreased proportion of antigen-inexperienced, naïve T cells is a hallmark of aging in both humans and mice, and contributes to reduced immune responses, particularly against novel and re-emerging pathogens. Naïve T cells depend on survival signals received during their circulation among the lymph nodes by direct contacts with stroma, in particular fibroblastic reticular cells. Macroscopic changes to the architecture of the lymph nodes have been described, but it is unclear how lymph node stroma are altered with age, and whether these changes contribute to reduced naïve T cell maintenance. Here, using 2-photon microscopy, we determined that the aged lymph node displayed increased fibrosis and correspondingly, that naïve T-cell motility was impaired in the aged lymph node, especially in proximity to fibrotic deposition. Functionally, adoptively transferred young naïve T-cells exhibited reduced homeostatic turnover in aged hosts, supporting the role of T cell-extrinsic mechanisms that regulate their survival. Further, we determined that early development of resident fibroblastic reticular cells was impaired, which may correlate to the declining levels of naïve T-cell homeostatic factors observed in aged lymph nodes. Thus, our study addresses the controversy as to whether aging impacts the composition lymph node stroma and supports a model in which impaired differentiation of lymph node fibroblasts and increased fibrosis inhibits the interactions necessary for naïve T cell homeostasis.

## Introduction

Older adults (approximately 65 years-old and older) have increased risk of poor outcomes against infectious disease (Simon et al., 2015). Implicated in declining health with age is the T cell compartment; the function and maintenance of T cells are profoundly impacted by age, and their alterations have been well-described (Nikolich-Žugich 2014; Goronzy and Weyand 2019). Among the changes to the T cell compartment is the expansion of memory T cells, which is accompanied by a decline in naïve T cells frequency and numbers. The proportion of naïve T cells contracts dramatically with age, with the frequency of CD4^+^ T cells reduced by half, and that of CD8^+^ T cells reduced seven-fold, when comparing patients between the ages of 18 and 80 years (Fagnoni et al., 2000). Likewise, the frequency of naïve T cells in circulation is also dramatically diminished in aged mice (Thompson et al., 2019). As the threat of emerging and re-emerging diseases increases in our global society (Morens et al., 2004), and given the critical role of naïve T cells in primary responses against novel pathogens (Appay and Sauce 2014), understanding the mechanisms that drive the deterioration of the naïve T cell compartment is key to improving aged immunity.

Early in life, output from the thymus bolsters naïve T cell numbers, but with thymic involution, starting shortly before puberty (approximately 10 years of age) for humans and around 8 weeks of age in mice (Srinivasan et al., 2021), naïve T cells are less augmented by the influx of *de novo* thymocytes but rather rely increasingly on homeostatic maintenance of existing naïve T cell clones (Goronzy et al., 2015). Naïve T cells are quiescent, long-lived, and depend on tonic signaling from the lymph nodes and spleen through major histocompatibility complex (MHC) molecules presenting self-antigens, as well as pro-survival cytokines such as interleukin-7 (IL-7). These T cell maintenance cues are sourced from the heterogeneous stromal cells of the secondary lymphoid organs; the fibroblast reticular cell (FRC) in particular has been demonstrated to be critical in its ability to support naïve T cell survival (Link et al., 2007; Martin et al., 2017). FRCs are non-hematopoietic stromal cells that serve as the structural integrity of the lymph nodes (Brown and Turley 2015; Pradhan et al., 2021). Subsets of FRCs are found in the cortex and medulla of the lymph nodes and are stellate or elongated in morphology, enabling them to form a reticular network (Katakai et al., 2004). The mechanical properties of FRCs are significant to their function, as relaxation of the FRC network is essential for lymphocyte expansion during immune activation (Astarita et al., 2015). T cells travel upon the FRC network in order to encounter antigen-presenting cells, and also obtain tonic T cell receptor signaling and cytokines from the FRCs themselves in order to promote T cell survival (Bajénoff et al., 2006).

Recent technological advances have enabled in-depth study of FRCs, revealing their important immunomodulatory roles (Perez-Shibayama et al., 2019). FRCs develop from mesenchymal stem cell progenitors, which interact with lymphoid tissue inducer (LTi) cells during embryonic development in order to form the lymph node anlagen (Bénézech et al., 2010). Committed progenitors, known as lymphoid tissue organizer (LTo) cells, differentiate into FRCs in a lymphotoxin-beta (LTβ)-dependent manner (Bénézech et al., 2010). Inducible deletion of *Ltbr* in adult mice resulted in loss of lymph nodes and Peyer’s patches (Shou et al., 2021), indicating that receptor activity remains important in the postnatal period. LTβ signals appear to be important for enforcing the immunoregulatory function of FRCs in the postnatal lymph node, as FRC-specific ablation of lymphotoxin-beta receptor (LTβR) resulted in poor pathogen clearance (Chai et al., 2013). Thus, FRCs depend on signaling through LTβR throughout adult life to drive the differentiation of mature effectors from stromal progenitors *in situ* and to maintain the immune-supportive functions of the lymph node.

The lymph nodes are regional centers of immune coordination, and their gross alteration with age would suggest some negative impact on their function, in keeping with the effect of aging on the primary lymphoid organs (Chinn et al., 2012). Aged lymph nodes have decreased size of the T cell zone and diminishment of the T cell zone:B cell follicle interface (Turner and Mabbott 2017), and furthermore fail to expand in size upon immune activation (Richner et al., 2015). Aged lymph nodes in mice and non-human primates also exhibit increased fibrosis (Thompson et al., 2019), mirroring what is observed in humans (Hadamitzky et al., 2010). Changes to FRCs themselves have been less obvious, as some studies have suggested they are decreased (Becklund et al., 2016), similar to young (Masters et al., 2019), or increased (Turner and Mabbott 2017) in proportion in aged mice. It is therefore unresolved whether FRCs are altered with age, and if age-associated changes could impair their role in maintaining naïve T cell homeostasis. In this study, we analyze the lymph node stroma of 16–20 month-old aged mice, which corresponds to approximately 55–65 year-old humans (Flurkey et al., 2007). Visualization using live-cell 2-photon microscopy revealed confined migratory spaces for naïve T cells within the aged lymph node, and that the T cells became enmeshed in fibrotic regions. Increased fibrosis was also associated with decreased *Ltbr* and *Ccl21* expression in aged stroma. Quantification of lymph node stroma revealed site-specific differences in FRC proportions, and FRC subsetting revealed reduced proportions of immature precursors. Thus, our studies suggest impaired LTβ-dependent maturation of FRCs within a context of extensive fibrosis, which supports a model in which the architecture of the lymph node prevents crucial FRC:lymphocyte contacts necessary for naïve T cell survival and generation of a robust immune response.

## Materials and Methods

### Mice

C57BL/6J, B6.SJL-Ptprca PepCb (CD45.1), and CAG-EGFP (Okabe et al., 1997) were bred and maintained under specific pathogen-free conditions in the Mayo Clinic animal facility. All strains were sourced from Jackson Laboratories, including aged C57BL/6J mice, in this study 16–20 months old. To reduce sex-related variability (Klein and Flanagan 2016), aged mice in the study were all female. Aged mice were monitored and health status visually assessed, and mice were removed from the study if in declining health. Mouse maintenance and experimental procedures were carried out with approval from the Institutional Animal Care and Use Committee at the Mayo Clinic.

### Live-Cell Two-Photon Microscopy

CD8^+^ naïve T cells were enriched from CAG-EGFP mouse spleens using EasySep Mouse Naïve CD8^+^ T cell isolation Kit (Stem Cell 19858) per the manufacturer’s instructions. CD8^+^ naïve T cells were confirmed to be >98% pure by flow cytometry. ∼10^6^ cells in 100 μL of PBS were injected retro-orbitally into B57BL/6 mice 24 h before imaging. Inguinal lymph nodes for imaging were dissected and embedded in 4% (w/v) NuSieve GTG low-melting-temperature agarose (Lonza) in PBS at 37°C. The solidified agarose block was sectioned into 300-μm-thick lymph node slices using a VT1200S Microtome (Leica) in a bath of ice-cold PBS, with speed at 0.20 mm s^−1^ and amplitude at 0.6 mm (Lancaster and Ehrlich 2017). Slices were collected in PBS + 10% FBS and kept on ice until ready for imaging.

Lymph node slices were transferred and secured in an imaging chamber (Harvard Apparatus) on the microscope stage. Perfusion medium, consisting of phenol red-free RPMI (Gibco) supplemented with 2 g L^−1^ sodium bicarbonate, 5 mM HEPES, and 1.25 mM calcium chloride, was circulated through the imaging chamber using a micro-perfusion high-flow pump (Bioptechs). The perfusion medium was aerated with 95% O_2_/5% CO_2_ and maintained at 37°C with a heated microscope stage and inline perfusion heater (Harvard Apparatus). Images were acquired every 15 s, through a depth of 40 μm, at 5-μm intervals for durations of 15–20 min, using an Investigator microscope (Bruker) with a 20× water immersion objective (Olympus, NA 1.0) and PrairieView software (v.5.5, Bruker). The sample was illuminated with a Chameleon Discovery NX ultrafast pulsed laser (Coherent) tuned to 920 nm. Emitted light was passed through 460/50 and 525/50 band-pass filters (Chroma) to separate GaAsP detectors for detection of second-harmonic generation (SHG, blue) and EGFP (green) fluorescence, respectively.

Migratory paths for T cells were tracked, and mean cell velocity and path straightness for each cell calculated using Imaris (v9.8, Bitplane). SHG signal was rendered as collagen *via* Surfaces. Distance Transformation function in ImarisXT was used to calculate distances between T cells and collagen Surfaces for every time point, and T cell Tracks were filtered as being proximal to collagen if the minimum Track distance was less than 12 μm at any time point.

### Immunofluorescence of Lymph Node Cryosections

Inguinal, axillary, brachial, cervical, and mesenteric lymph nodes from mice were harvested and immediately submerged in 1% paraformaldehyde in PBS overnight and fixed overnight in 20% sucrose (w/v) in PBS (Choi et al., 2020). Fixed lymph nodes were embedded in Optimal Cutting Temperature (OCT) compound (Ted Pella) within TissueTek Cryomolds (VWR) and snap frozen using liquid nitrogen or isopentane with dry ice. Frozen tissue blocks were then sectioned on a HM525NX Cryostat (Thermo Scientific) at 16-μm thickness at −15°C onto poly-L-lysine-coated microscope slides (VWR).

To stain, defrosted slides were fixed for 20 min in ice-cold acetone then washed in PBS and PBS-T (PBS + 0.1% Tween-20). Sections were blocked for 15 min in TNB blocking buffer [0.1 M Tris-HCl, pH 7.5; 0.15 M NaCl; and 0.5% (w/v) blocking reagent (Perkin Elmer FP1020)]. Sections were stained overnight at 4°C with the following primary antibodies diluted in TNB blocking buffer: rabbit polyclonal α-mouse alpha-smooth muscle actin (1:200, Abcam 32575), rabbit polyclonal α-mouse collagen IV (1:200, Abcam 6586), rabbit polyclonal α-PDGFRβ (1:200, Abcam 6586), rat α-mouse/human B220 (1:400, RA3-6B2, Biolegend 103201), Syrian hamster α-mouse gp38/podoplanin (1:200, 8.1.1, Biolegend 127401). Sections were washed with PBS-T before incubation for 1 h at RT with secondary antibodies diluted (1:200) in TNB blocking buffer: AF488 goat α-rat IgG (1:400 dilution; Biolegend Poly4054), AF488 goat α-Syrian hamster IgG (Jackson Immunoresearch 107-545-142), AF594 donkey α-rabbit IgG (Invitrogen A32754), Cy3 donkey α-rabbit IgG (Jackson Immunoresearch 711-165-152). Nuclei were stained with 3 μM DAPI and coverslips sealed with Prolong Gold antifade reagent (Sigma Aldrich). Sections were imaged using a LSM 800 confocal microscope (Zeiss) equipped with Plan-Neofluar 20× objective lens (Zeiss, NA 0.4). Quantitative comparison of αSMA fluorescence density was conducted using Imaris on 20× magnification images. Threshold for αSMA^hi^ was set based on aged lymph node samples, and used to render Surfaces. Total volume of Surfaces was calculated as a percent of total field of view.

### Isolation of Lymph Node Stromal Cells

Inguinal, axillary, brachial, superficial cervical, and mesenteric lymph nodes from mice were dissected into plastic cell culture dishes containing phosphate buffered saline (PBS). Excess fat was trimmed away using surgical scissors under a dissection stereomicroscope. Lymph nodes were enzymatically digested into single cell suspension as follows (Fletcher et al., 2011; Knop et al., 2020): lymph nodes were cut in half using surgical scissors and incubated at 37°C, 5%CO_2_ for 30 min in separate wells of a twelve-well plate with 1 ml each of an enzyme digest cocktail, which consisted of complete RPMI 1640 medium [RPMI 1640 (Corning) and 10% fetal bovine serum (Gibco), supplemented with 100 U/mL penicillin-streptomycin (Thermo Fisher), non-essential amino acid solution (Sigma Aldrich), 1 mM sodium pyruvate, GlutaMAX (Thermo Fisher), and β-mercaptoethanol (Sigma Aldrich)] with 0.2 mg/ml of Dispase II (Sigma D4693), 0.4 mg/ml Collagenase P (Roche 11213857001), and 0.010 mg/ml of DNAse I (Roche 04536282001)]. Another 1 ml of the enzyme digest cocktail was added to each well and the well contents gently agitated by aspiration using a glass pipette before a second 30 min incubation. The tissue was completely dissociated by more aggressive aspiration using a glass pipette, and the digest was quenched by addition of 0.5 ml of complete RPMI 1640 medium containing 10 mM EDTA and transferred through 100-μm mesh filters.

Lymph node stroma were enriched in the young and aged samples by depletion of CD45^+^ hematopoietic cells using magnetic bead-based cell separation as follows (Fletcher et al., 2011): samples were centrifuged and resuspended in 90 μL of stromal wash buffer (2% FBS in PBS with 5 mM EDTA) per 10^7^ cells. 7 μL of CD45 Microbeads (Miltenyi Biotech 130-052-301) were added per 10^7^ cells and incubated on ice for 20 min. The samples were washed and resuspended in stromal wash buffer. CD45^+^ cells were collected by passing the samples through LD Columns (Miltenyi Biotech 130-042-901) supported by a QuadroMAX Separator (Miltenyi Biotech 130-090-976). Column flow-through was collected as enriched CD45^−^ lymph node stroma. Stroma was confirmed to be >98% CD45^−^ by flow cytometry.

### Flow Cytometric Analysis and Sorting of Stromal Subsets

Up to 10^7^ cells were incubated for 20 min on ice in 100 μL of PBS + 2% FBS with fluorochrome-conjugated antibodies directed against the following mouse targets: CD3-PECy5 (145-2C11, Biolegend 100309), CD19-PECy5 (6D5, Biolegend 115509), CD31-APCCy7 (390, Biolegend 102439), CD35-AlexaFluor700 (7E9, Biolegend 123431), CD45-PECy5 (30-F11, Biolegend 103109), B220-BV711 (RA3-6B2, Biolegend 103255), gp38-FITC (8.1.1, Biolegend 127415), ICAM-1-BV421 (YN1/1.7.4, Biolegend 116141), LTBR-PECy7 (5G11, Biolegend 134409), MAdCAM-1-APC (MECA-367, Biolegend 120711), SCA-1-BV605 (D7, Biolegend 108133), VCAM-1-PE (429 (MVCAM.A), Biolegend 105713). Antibodies were diluted (1:200) from stock concentrations of 0.5 mg ml^−1^. Cells were washed and resuspended in 10 μg ml^−1^ propidium iodide (PI) to determine viability. Samples were analyzed on an LSR Fortessa or Symphony flow cytometer (BD Biosciences) and data were analyzed using FlowJo (v.10, TreeStar).

For stromal subset sorting, pooled CD45-depleted cells from lymph nodes were incubated for 20 min on ice in 100 μL of PBS + 2% FBS with fluorochrome-conjugated antibodies directed against the following mouse targets: CD31-APC (MEC13.3, Biolegend 102509), CD35-APCCy7 (7E9, Biolegend 123417), CD45-APCCy7 (30-F11, eBioscience 47-0451-82), gp38-FITC (8.1.1, Biolegend 127415), and ICAM-1-BV421 (YN1/1.7.4, Biolegend 116141). Antibodies were diluted (1:200) from stock concentrations of 0.5 mg ml^−1^. Cells were washed and resuspended in 10 μg ml^−1^ PI to determine viability. Samples were sorted on an Aria FACS (BD Biosciences) based on the following criteria: fibroblastic reticular cells (FRCs; CD45^−^CD35^−^ICAM-1^+^gp38^+^CD31^−^), lymphatic endothelial cells (LECs; CD45^−^CD35^−^ICAM-1^+^gp38^+^CD31^+^), blood endothelial cells (BECs; CD45^−^CD35^−^ICAM-1^+^gp38^−^CD31^+^), and double-negative cells (DNs; CD45^−^CD35^−^ICAM-1^+^gp38^−^CD31^−^). Samples were confirmed to be >95% pure.

### Adoptive Transfer of Naïve T Cells

Spleens were dissected from 2-3-month-old CD45.1 mice and mechanically dissociated into a single cell suspension. Splenocytes were labelled with CellTrace Violet (Biolegend C34571), and CD4^+^ naïve T cells enriched using the EasySep Mouse Naïve CD4^+^ T cell isolation kit (StemCell), all per manufacturers’ instructions. CD4^+^ naïve T cells were confirmed to be >95% pure by flow cytometry. 2 × 10^6^ naïve T cells were adoptively transferred *via* intravenous injection into sub-lethally irradiated (600 cGy in split doses) 8-week-old and 17–20-month-old C57BL/6J recipient mice. After 6 days, spleens and lymph nodes were dissected and pooled from recipients. Up to 10^7^ cells were incubated for 20 min on ice in 100 μL of PBS + 2% FBS with fluorochrome-conjugated antibodies directed against the following mouse targets: CD3-APCCy7 (17A2, Biolegend 100222), CD4-BV510 (RM4-5, Biolegend 100553), CD8-BUV395 (53-6.7, BD Biosciences 563786), CD44-AF700 (IM7, Invitrogen 56-0441-82), CD45.1-PECy7 (A20, Biolegend 110730), CD45.2-AF700 (104, Biolegend 109822), and CD62L-BV605 (MEL-14, Biolegend 104437). Antibodies were diluted (1:200) from stock concentrations of 0.5 mg ml^−1^. Cells were washed and resuspended in 10 μg ml^−1^ PI to determine viability. For analysis of phosphorylated STAT5, cells were stained for surface markers and with fixable viability dye Zombie Red (Biolegend 77475) before fixation using FOXP3/Transcription Factor fixation kit (eBioscience), per manufacturer’s instructions, and intracellular staining with pSTAT5 (pY694)-FITC (BD Bioscience 612598) or isotype control (BD Phosflow mouse IgG1κ, BD Bioscience 557782). Antibodies were diluted (1:200) from stock concentrations of 0.5 mg ml^−1^. Samples were analyzed on an LSR Fortessa or Symphony flow cytometer (BD Biosciences) and data were analyzed using FlowJo (v.10, TreeStar).

### Naïve T Cell Survival Assay

Lymph nodes were dissected from 2-3-month-old and 16–20 month-old C57BL/6J mice and enzymatically digested as above. Stroma were cultured as follows (Link et al., 2007; Fletcher et al., 2011): 10^6^ lymphocytes in complete RPMI medium were plated in 96-well flat-bottom plates and incubated overnight at 37°C, 5% CO_2_. Non-adherent cells were gently removed by repeated washing with PBS, and the adherent stromal cells were again incubated in complete RPMI medium for 48 h.

Lymphocytes from 2-3-month-old CD45.1^+^ mice were mechanically disassociated into a single-cell suspension. 10^6^ lymphocytes were added to wells with stromal cells, along with fresh complete RPMI medium. In some wells, 0.1 ng ml^−1^ recombinant murine IL-7 (Cell Signaling) and/or 10 μg ml^−1^ α-IL-7 (M25, BioXCell BE0048), were added. The cells were incubated for 3 days before staining by incubation for 20 min on ice in 100 μL of PBS + 2% FBS with fluorochrome-conjugated antibodies directed against the following mouse targets: CD3-BUV395 (17A2, BD Biosciences 740268), CD4-APC (GK1.5, Biolegend 100412), CD8-Pacific Blue (53-6.7, Biolegend 100725), CD44-PE (IM7, Biolegend 103008), CD45.1-PECy7 (A20, Biolegend 110730), CD45.2-AF700 (104, Biolegend 109822), and CD62L-FITC (MEL-14, BD Pharmingen 553150). Cells were washed and resuspended in 10 μg ml^−1^ PI to determine viability. Cell numbers were calculated with the addition of 2 × 10^4^ polystyrene beads (Polysciences). Samples were analyzed on an LSR Fortessa or Symphony flow cytometer (BD Biosciences) and data were analyzed using FlowJo (v.10, TreeStar).

### Quantitative PCR

RNA from up to 10^6^ cells was purified using the RNeasy Mini Kit (Qiagen, 74104) according to manufacturer’s instructions, and RNA concentration was then measured by NanoDrop One (Thermo Scientific). cDNA was synthesized using SuperScript^®^ IV First-Strand Synthesis SuperMix (Invitrogen, 18091050) per the manufacturer’s instructions, on a C1000 Touch thermocycler (BioRad). Gene expression levels of *Ccl19*, *Ccl21*, *Il7*, *LIGHT*, *Lta*, *Ltb*, and *Ltbr* were measured relative to *Actb* using LightCycler 480 SYBR Green I Master (Roche, 04707516001) on an LightCycler 480 system (Roche) following the manufacturer’s instructions, using the following primers: *Actb*: forward- CAT​TGC​TGA​CAG​GAT​GCA​GAA​GG, reverse- TGC​TGG​AAG​GTG​GAC​AGT​GAG​G; *Ccl19a*: forward- CTG​CCT​CAG​ATT​ATC​TGC​CAT, reverse-AGGTAGCGGAAGGCTTTCAC (Chai et al., 2013); *Ccl21a*: forward- AAG​GCA​GTG​ATG​GAG​GGG​GT, reverse- CTT​AGA​GTG​CTT​CCG​GGG​TG (Chai et al., 2013); *Il7*: forward- CTG​ATG​ATC​AGC​ATC​GAT​GAA​TTG​G, reverse-GCAGCACGATTTAGAAAAGCAGCTT (Martin et al., 2017); *LIGHT*: forward- CAG​GCC​CCT​ACA​GAC​AAC​AC, reverse- ACT​CGT​CTC​CCA​CAA​GGA​ACT (Seach et al., 2008); *Lta*: forward- GCT​TGG​CAC​CCC​TCC​TGT​C, reverse- GAT​GCC​ATG​GGT​CAA​GTG​CT (Seach et al., 2008); *Ltb*: forward- CCA​GCT​GCG​GAT​TCT​ACA​CCA, reverse- AGC​CCT​TGC​CCA​CTC​ATC​C (Seach et al., 2008); *Ltbr*: forward-TGCTCTTCACCACTGTCCTG, reverse- GAA​ATG​TGG​GTC​GGC​TCT​T (Grandoch et al., 2015).

### Statistics

All statistical analyses were performed using Prism (v. 9, GraphPad) with the corresponding test listed in the Figure Legends.

## Results

### Reduced T Cell Migration in the Aged Fibrotic Lymph Node

**FIGURE 1:**
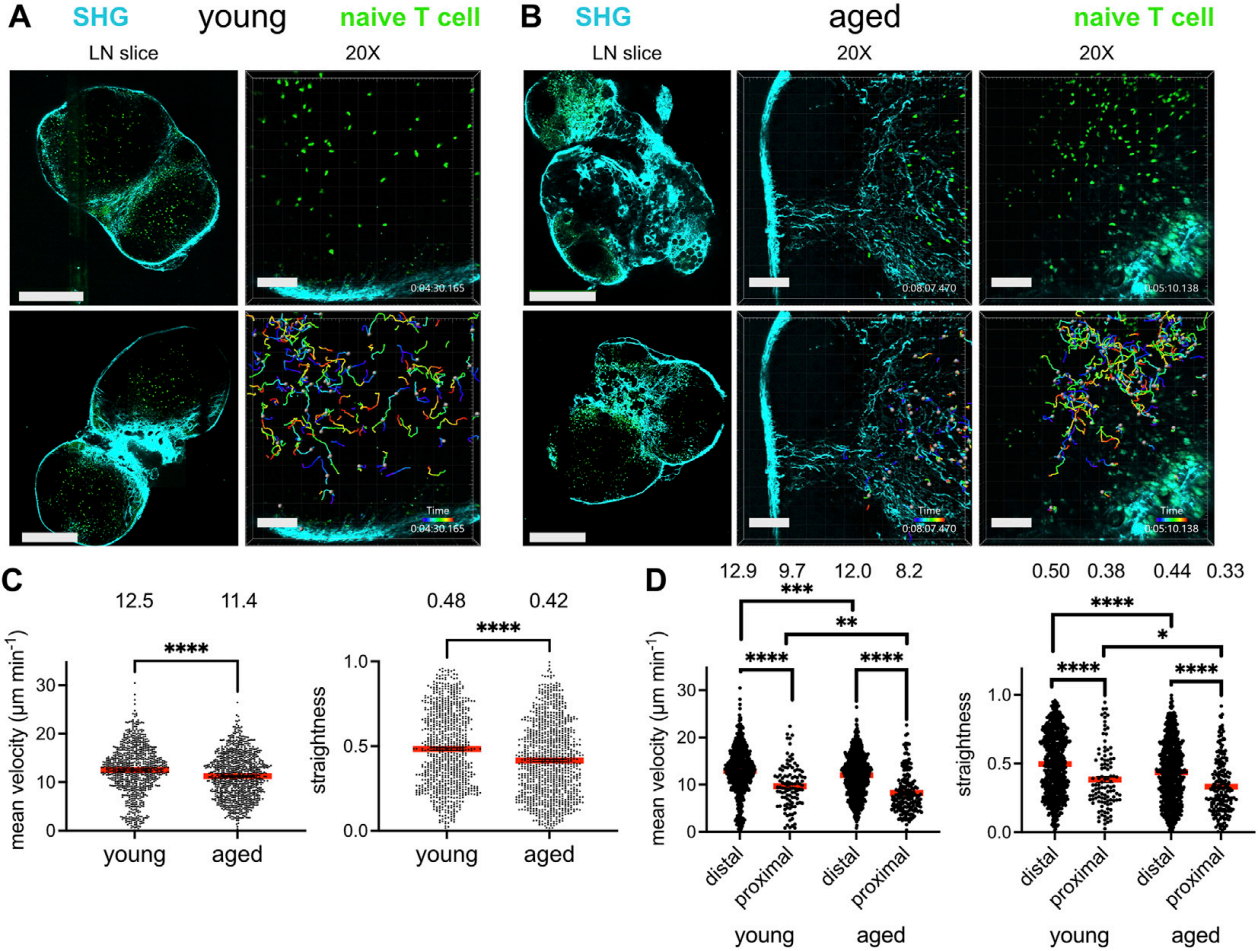
Reduced T cell migration in the aged fibrotic lymph node. Young (2-3MO) EGFP-expressing CD8^+^ naïve T cells were adoptively transferred into young (2-3MO) and aged (16-20MO) WT hosts 24 h before lymph nodes were harvested and prepared for live-cell explant 2-photon microscopy (2PM). Time-lapse imaging through 40-μm depth was carried out with laser tuned to 920 nm to simultaneously excite EGFP (green) and second harmonic generation (SHG, cyan). **(A)**. Imaging of young lymph node slices: 20× tile-scans of representative lymph node slices (left panels); Scale bar is 550 μm. Maximum intensity projections of 2PM volume at 20× magnification (right panels). Naïve T cell track times are color-encoded as indicated in bottom panel; Scale bar is 100 μm. **(B)**. Imaging of aged lymph node slices: 20× tile-scans of representative lymph node slices (left panels); Scale bar is 550 μm. Maximum intensity projections of 2PM volume at 20× magnification, with representative volumes containing increased (middle panels) or less fibrosis (right panels). Naïve T cell track times are color-encoded as indicated in bottom panels; Scale bar is 100 μm. **(C)**. Mean velocities (left panel) and track straightnesses (right panel) of individual naïve T cells migrating in young and aged lymph node slices. **(D)**. Mean velocities (left panel) and track straightnesses (right panel) of individual naïve T cells migrating in young and aged lymph node slices, categorized based on being distal (>12 μm) or proximal (≤12 μm) to collagen. Data points represent cells from three experiments, and red bars with numbers show mean values, number of cells on slices: young (*N*_distal_ = 712, *N*_proximal_ = 105), aged (*N*_distal_ = 748, *N*_proximal_ = 193). Analyzed by *t* tests, *p*-values: ns, not significant; * < 0.05, ** < 0.01, *** < 0.001, **** < 0.0001.

Given that T cells migrate within the lymph node to encounter antigen-presenting cells bearing their cognate antigen and to engage in survival crosstalk, we investigated whether the aged lymph node microenvironment could impact the ability of naïve T cells to efficiently navigate the lymph nodes. GFP-expressing naïve T cells, harvested from young (2–3 month-old) mice, were adoptively transferred into young and aged (16–20 month-old) C57BL/6J hosts 24 h prior to imaging. Lymph nodes were harvested and prepared as slices for immediate *ex vivo* imaging, a well-established technique for studying lymphocyte dynamics within an intact lymph node microenvironment (Bajénoff et al., 2006). Collagen is a birefringent material, and thus can be observed label-free by 2-photon microscopy *via* second harmonic generation (Bougherara et al., 2015). Indeed, young lymph nodes were encapsulated within a collagen capsule, with collagen also prominent in the medullary sinuses, but no collagen was observed within the T-cell zones. (Figure 1A, left panels). Aged lymph node slices, on the other hand, were much less consistent in their structure, with some regions bearing resemblance to the typical kidney bean-shaped form, but many other slices with an irregular outline. Such aged slices harbored varying degrees of encroachment by fibrosis into the T cell zones (Figure 1B, left panels). For aged slices with increased collagen signal and irregular shape, T cell zones were highly constricted, though aged slices with a more typical lymph node shape still retained identifiable T cell zones. GFP^+^ T cells were dispersed throughout expansive T cell zones that flank the medulla in young slices, and live imaging revealed that they migrate quickly and randomly (Figure 1A, right panels; Supplementary Movie S1). In some aged slices, collagen formed a mesh within the T cell zone, which severely restricted the motility of T cells (Figure 1B, middle panels; Supplementary Movie S2), but for slices in which collagen has not encroached too deeply, T cell velocity was high (Figure 1B, right panels; Supplementary Movie S3).

The mean T cell velocities and path straightesses were quantified between the young and aged slices. Naïve T cells traveling within aged lymph node slices had significantly reduced mean cell velocities than those on young slices (Figure 1C, left panel). The mean path straightness, a parameter which relates the overall length of the T cell path travels to total displacement, was also reduced for cells in aged slices (Figure 1C, right panel), indicating more restricted migration trajectories in the aged microenvironment. As the speed of T cell migration within aged slices appeared to depend on whether fibrosis was prominent in the tissue parenchyma, the data were stratified based on T cells that were distal from collagen fibers and those that were proximal to collagen, within one cell-length (<12 μm). The mean velocity of T cells migrating distal to collagen were significantly greater than those proximal to collagen in both lymph node ages (Figure 1D, left panel). T cells migrating proximal to collagen were significantly slower in both tissues, with mean average velocities of ∼8–9 μm min^−1^ (Figure 1D, left panel). However, T cells far from collagen in young slices were still faster than those far from collagen in aged slices, as were T cells close to collagen (Figure 1D, left panel). In young slices, ∼12% of T cells were near collagen fibers, whereas ∼20% of T cells were near collagen fibers in aged slices, indicating that a greater proportion of T cells were slowed in the aged microenvironment. For both ages of lymph node slices, T cells traveled with reduced path straightnesses when proximal to collagen, indicating confined trajectories with little displacement (Figure 1D, right panel). However, T cells both distal and proximal to collagen sources in aged slices traveled in a more confined pattern than their counterparts in young slices (Figure 1D, right panel). Thus, our imaging studies demonstrated that young naïve T cells traveled with less velocity and straightness when placed in an aged rather than a young lymph node microenvironment. Decreased motility was associated with increased deposition of fibrosis into the T cell zone of aged lymph nodes slices, which severely limited the motility of T cells.

### Increase Fibrosis in the Aged T Cell Zone Network

**FIGURE 2:**
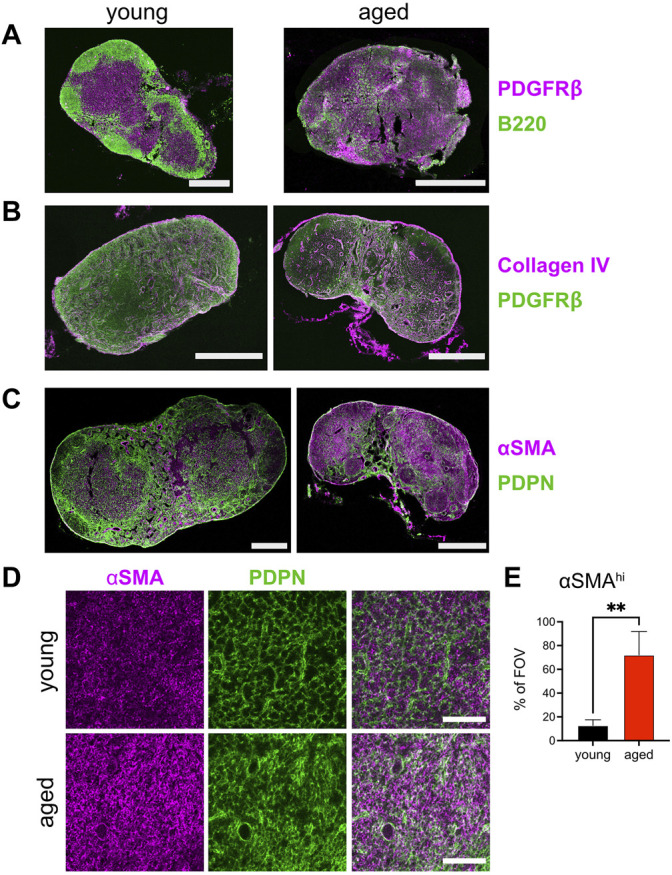
Increase fibrosis in the aged T cell zone network. Lymph nodes from young (2-3MO) and aged (16-20MO) WT mice were harvested, fixed, and frozen for cryosectioning. Confocal 20× tile-scans of lymph node cross-sections stained with antibodies directed against **(A)**. PDGFRβ (magenta) and B220 (green), **(B)**. collagen IV (magenta) and PDGFRβ (green), **(C)**. αSMA (magenta) and PDPN (green) in young and aged mice. Scale bar is 500 μm. **(D)**. Maximum intensity projection of 12-μm thick confocal volumes, at 20× magnification, stained for αSMA (magenta) and PDPN (green) in young and aged mice. Scale bar is 100 μm. Data is representative of lymph nodes retrieved from 6 young and 6 aged mice. **(E)**. Percentage of αSMA^hi^ voxels within T cell zone confocal volumes (as in D) in young and aged lymph node fields of view (FOV). Analyzed by *t* test, *p*-value: ** < 0.01.

As aging in mouse models is often studied at >20 months, we confirmed that structural dysregulation of lymph node compartments occurred at our relatively earlier time points of ≥16 months. In young adult mice, we observed fine compartmentalization of B cell follicles in the subcapsular region from the T cell zone paracortex, by staining B cells using an antibody directed against B220 and platelet-derived growth factor-beta^+^ (PDGFRβ^+^) fibroblasts (Figure 2A, left panel). With age, the lymph node was rounded and compacted, as in our 2-photon imaging, and B cell areas were no longer definable as follicles (Figure 2A, right panel). Rather, PDGFRβ staining was now spread throughout the lymph node and mixed with B220^+^ areas, though its PDGFRβ staining pattern was yet diffuse when compared to the T cell zone of the young lymph node. Thus, aged murine lymph nodes have lost the compartmentalization of the B cell follicles by 16 months of age.

To confirm the SHG fluorescence of fibrosis observed by 2-photon microscopy, we co-stained for collagen IV and PDGFRβ in young and aged lymph node sections. Collagen IV staining the capsular sheath and perivascular cross-sections within the young lymph nodes (Figure 2B, left panel). In the aged lymph node, the capsule was also intensely stained for collagen IV; reticular staining in the paracortex and staining of collagen enwrapping the vessels were more prominent than visualized in young lymph nodes (Figure 2B, right panel). We further stained for alpha-smooth muscle actin (αSMA) against the fibroblast marker podoplanin (PDPN; gp38). Like PDGFRβ, αSMA is a mesenchymal fibroblast specific marker (Chai et al., 2013; Choi et al., 2020). PDPN is expressed by FRCs and lymphatic endothelial cells (LECs), and is a glycoprotein responsible for the contractility of lymph node stroma (Astarita et al., 2015). In the young lymph node, PDPN stains a fine network through the paracortex and in the medullary sinuses, with αSMA interspersed between PDPN^+^ reticular cells of the T cell zone and most prominent in the lining of vessels of the medulla (Figure 2C, left panel and Figure 2C, top row). Though captured at the same settings as the young lymph nodes, the aged lymph node sections stained more intensely for αSMA (Figure 2C, right panel and Figure 2D, bottom row). While the SHG signal captured by 2-photon microscopy emphasized fibers in the subcapsular and medullary areas, staining for αSMA was most dense in the cortical regions. Closer observation of the PDPN channel in the cortex reveal that the network is still maintained, albeit with increased density that is thoroughly intercalated with αSMA (Figure 2D, bottom row, and Figure 2E). Thus, immunofluorescence for fibroblast-associated markers indicated increased deposition of collagen and αSMA within the architecture of the aged lymph node.

### Naïve T Cell Survival is Impaired *in vivo* but not *in vitro*

**FIGURE 3:**
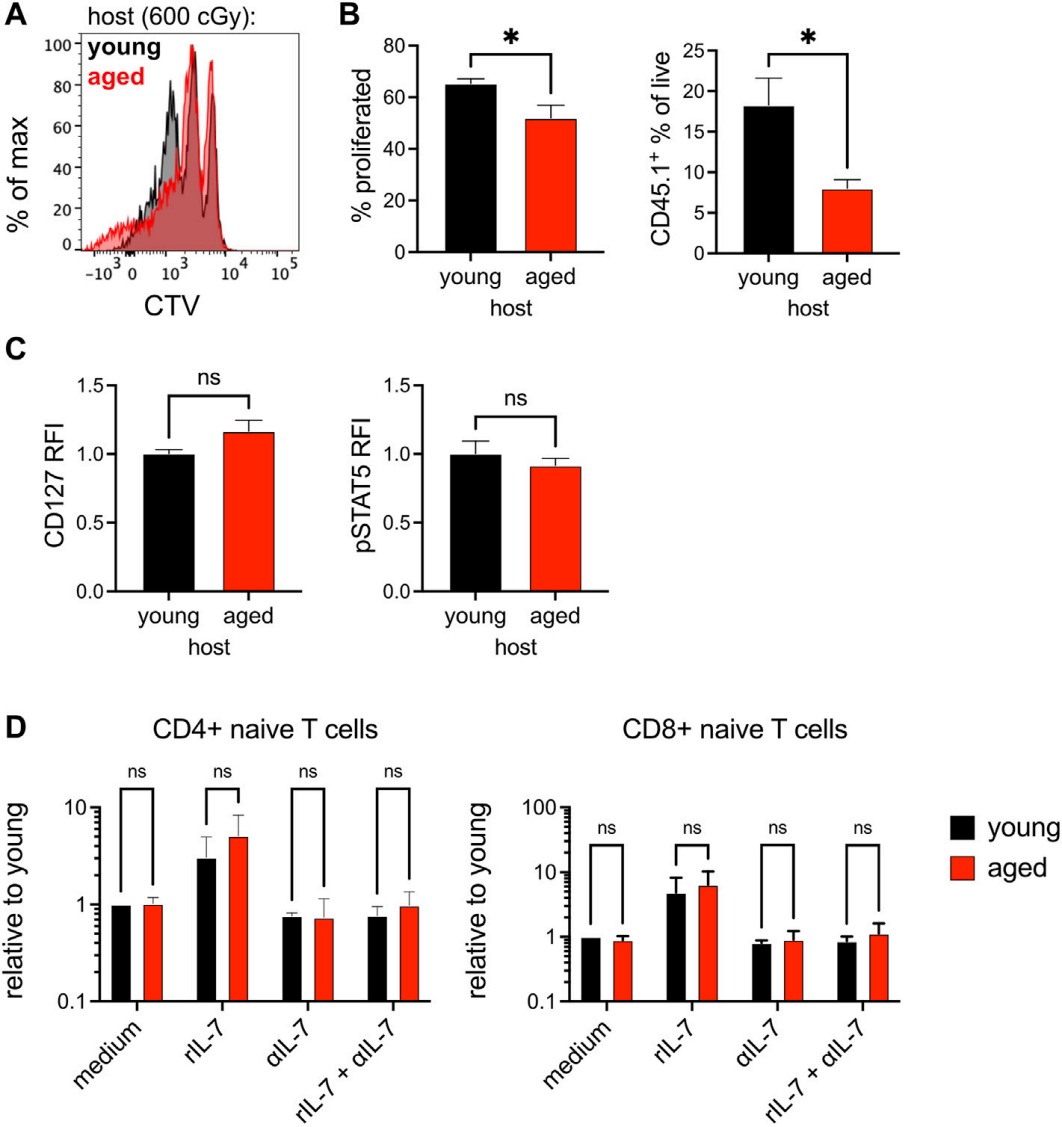
Naïve T cell survival is impaired *in vivo* but not *in vitro.* Cell Trace Violet (CTV)-labeled CD4^+^ naïve T cells isolated from young CD45.1^+^ mice were adoptively transferred into sublethally-irradiated young and aged WT hosts. **(A)**. Histogram of CTV dilution in CD45.1^+^CD4^+^CD62L^hi^CD44^lo^ cells in young (black) and aged (red) hosts. **(B)**. The percent of naïve CD45.1^+^ T cells that have diluted CTV during proliferation (left panel) and the percent of live host cells derived from the CD45.1^+^ donor (right panel). Relative fluorescence intensity (RFI) of **(C)**. CD127 and pSTAT5 in adoptively-transferred naïve T cells in young (black) and aged (hosts). Compiled data from two experimental trials shown, with each trial consisting of two age groups of three recipients. RFI was based on normalization to the mean of the cells within the young host for the respective experimental trial. Analyzed by *t* tests on compiled data, *p*-values: ns, not significant; * < 0.05. **(D)**. Lymphocytes from young CD45.1^+^ mice were added to established cultures of young or aged lymph node CD45^−^ stromal cells, with or without supplementation with rIL-7 and α-IL-7. Numbers of congenic naïve CD4^+^ and CD8^+^ T cells was quantified after 3-day co-culture. Compiled data from three experimental trials shown, with each trial consisting of triplicate wells and cell numbers normalized to the mean of cells present when co-cultured with young stroma. Means from each experiment were analyzed by two-way ANOVA with adjustment for multiple comparisons, *p*-values: ns, not significant.

Our 2-photon imaging demonstrated that T cell motility was impaired in the aged lymph node, especially when in proximity to collagen deposition; however, there was no obvious change in the capacity for young naïve T cells to home to the aged lymph nodes, and many T cells were able to migrate at high velocity when far from collagen fibers. To validate our aged mouse model as a context in which naïve T cell survival was impaired, we recapitulated experiments conducted by Becklund and colleagues to confirm that naïve T cell homeostatic proliferation was indeed decreased in aged mice (Becklund et al., 2016). Cell Trace Violet (CTV)-labelled congenic CD4^+^ naïve T cells from young mice were adoptively transferred into young and aged mice. Hosts were sub-lethally irradiated before adoptive transfer so that T cells would undergo non-activating homeostatic proliferation in order to repopulate T cell numbers. Naïve T cells diluted CTV in response to homeostatic proliferation to a greater extent when place within young rather than aged hosts, indicating that they underwent more rounds of homeostatic proliferation (Figures 3A,B). Notably, the proportion of transferred young naïve T cells detected in young hosts was also significantly greater than that in aged hosts (Figure 3B). To determine whether changes in homeostatic proliferation were due to differences in signaling through IL-7 receptor, we measured the mean fluorescence intensity (MFI) of CD127 (IL-7Rα chain) in the transferred cells, as the receptor is internalized in response to IL-7 signaling (Martin et al., 2017). However, we did not measure any significant difference in the MFI of CD127 for the congenic naïve T cells within young or aged hosts (Figure 3C, left panel), nor did we measure any significant difference in T cell phosphorylation of STAT5, downstream of IL-7R signaling (Figure 3C, right panel). Thus, homeostatic proliferation of young naïve T cells was impaired within the context of the aged microenvironment, but not associated with any obvious differences in IL-7 signaling.

In order to determine if decreased homeostatic proliferation observed *in vivo* was due to cell-specific changes to aged stroma, we adopted a well-established lymph node stroma co-culture assay (Link et al., 2007; Fletcher et al., 2011). Young congenic lymphocytes were incubated with CD45-depleted young or aged lymph node stromal cells, and the survival of naïve T cells was measured after 3 days. We measured no significant difference in the survival of naïve CD4^+^ or CD8^+^ T cells when co-cultured with stroma from young or aged lymph nodes (Figure 3D). Addition of a low concentration of recombinant murine IL-7 (rIL-7) significantly increased naïve T cell numbers when co-cultured with stroma, and increased survival was negated by inclusion of IL-7-blocking antibody (αIL-7) (Figure 3D). We concluded from our *in vitro* assays that bulk aged stroma do not have an inherent defect in supporting naïve T cells.

### Reduced Expression of *Ltbr* and *Ccl21* in Aged Stroma

**FIGURE 4:**
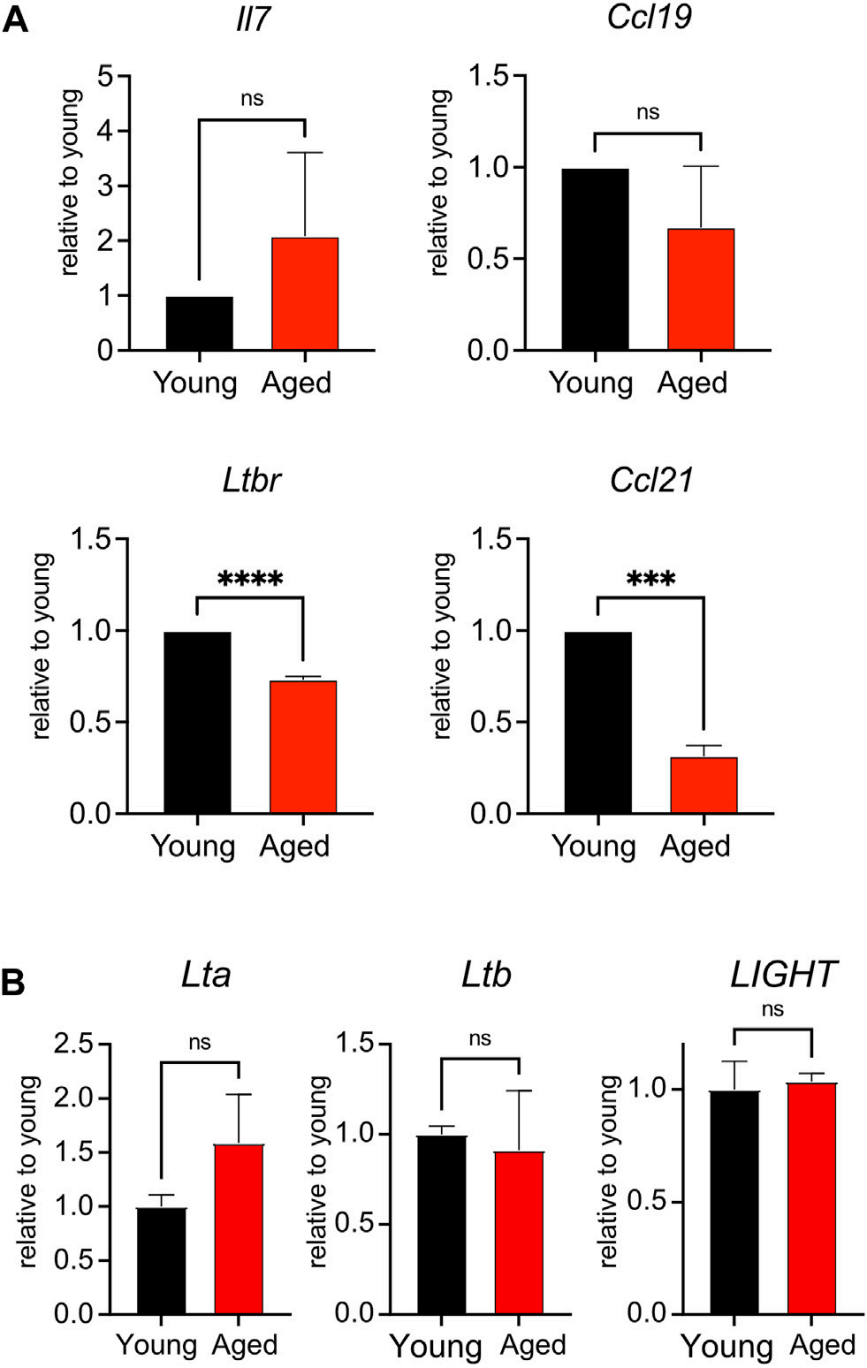
Reduced expression of *Ltbr* and *Ccl21* in aged stroma. **(A)**. Lymph nodes from young and aged WT mice were depleted of CD45^+^ cells before qPCR measurement of *Il7*, *Ltbr*, *Ccl19*, and *Ccl21* expression. Data were normalized to *Actb* expression for each mouse, then normalized to young transcript expression for the given experiment. Compiled data from 3 experiments, in which lymph node stroma from 3 young and 3 aged mice was pooled for each experiment. Analyzed by *t* tests, *p*-values: ns, not significant; *** < 0.001, **** < 0.0001. **(B)**. Splenocytes from young and aged mice were analyzed by qPCR for *Lta*, *Ltb*, and *LIGHT* expression. Data were normalized to *Actb* expression for each mouse, then normalized to young transcript expression for the given experiment. Compiled data from 2 experiments, analyzed by *t* tests: ns, not significant.

To determine whether aging impacted the homeostatic function of the lymph node stroma, we surveyed the global expression of *Il7*, a T cell maintenance cytokine, and chemokine ligands *Ccl19* and *Ccl21*, which promote T cell homing to and interactions within the secondary lymphoid organs (Link et al., 2007). We furthermore quantified stromal expression of *Ltbr*, as signaling through this receptor enforces development of lymph node stroma (Bénézech et al., 2010; Chai et al., 2013). We found no significant difference in the expression of *Il7* or *Ccl19* when comparing young and aged stroma (Figure 4A). However, we found significant decreases in the aged stromal expression of *Ccl21* and *Ltbr* (Figure 4A). Both *Ltbr* and *Ccl21* were highly expressed in FRCs from sorted adult stroma (Supplementary Figure S1), suggesting that the FRC population may play a predominant contribution to the age-associated decline observed. Since *Ltbr* expression was decreased with age, we measured the expression of lymphotoxin ligands *Lta*, *Ltb*, and *LIGHT* in bulk splenocytes from young and aged mice. As stroma constitute <1% of the cellularity of the secondary lymphoid organs, they would have a negligible impact in the measurement of these ligands in hematopoietic cells. We found no significant difference in the expression of *Lta*, *Ltb*, and *LIGHT* between young and aged cells (Figure 4B). This indicated that decreased *Ltbr* expression by aged stroma was not associated with changes in ligand expression. Thus, we determined that aging was associated with decreased transcript expression of *Ltbr* in stroma, and the homeostatic chemokine *Ccl21*.

### Reduction of Fibroblast Reticular Cell Progenitors in the Aged Lymph Nodes

**FIGURE 5:**
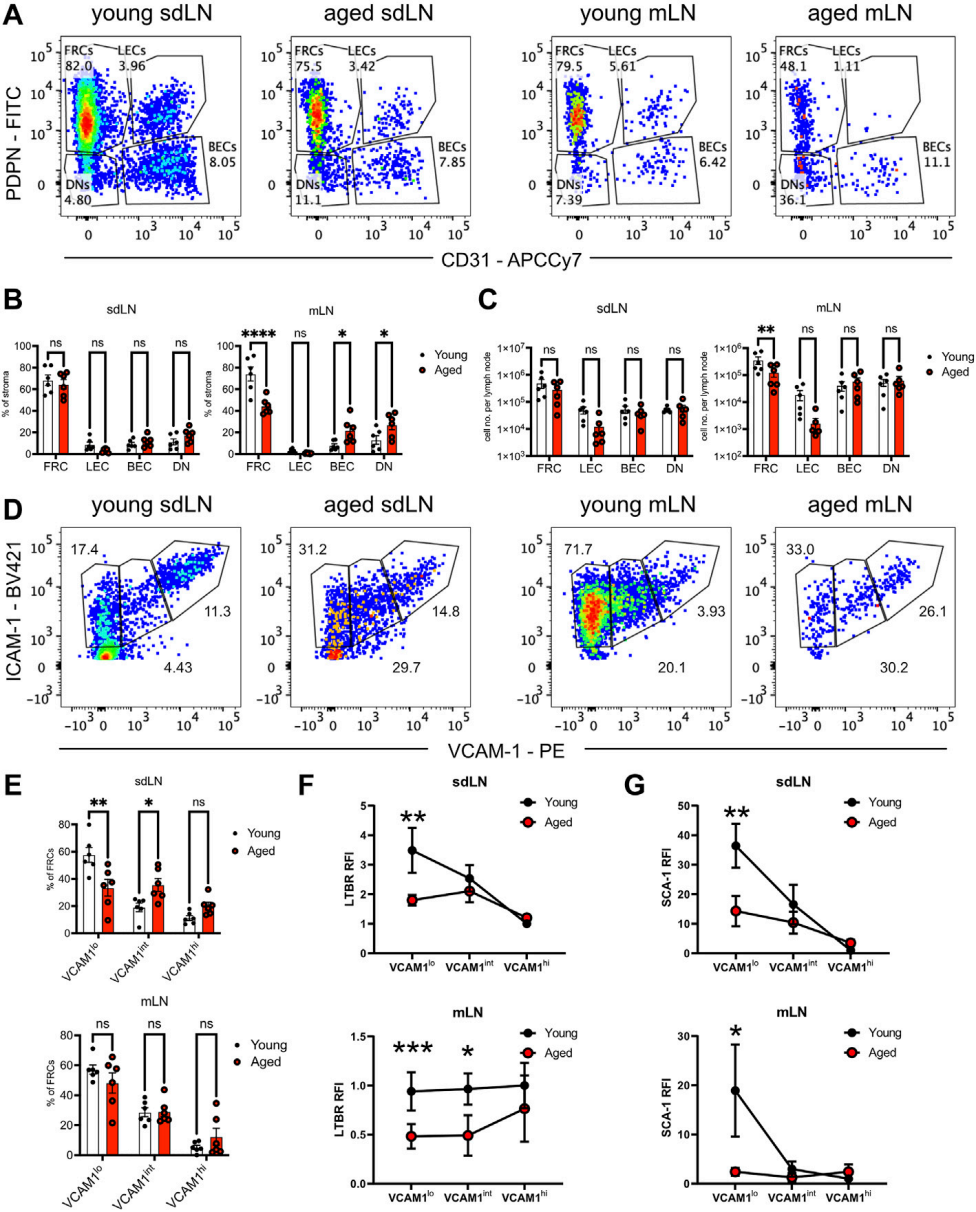
Reduction of FRC progenitors in the aged lymph nodes. Skin-draining (sdLN) and mesenteric (mLN) lymph nodes from young and aged WT mice were depleted of CD45^+^ cells before analysis by flow cytometry. **(A)**. Gating of CD45^-^CD35^-^B220^-^ICAM-1^+^ stroma into fibroblastic reticular cells (FRCs), lymphatic endothelial cells (LECs), blood endothelial cells (BECs), and double negative cells (DNs) in the sdLN and mLN of young and aged mice. **(B)**. Frequency of FRCs, LECs, BECs, and DNs within young and aged sdLN and mLN stroma. **(C)**. Number per lymph node of FRCs, LECs, BECs, and DNs within young and aged sdLN and mLN stroma. **(D)**. Gating of MAdCAM-1^-^ FRCs into VCAM-1^lo^, VCAM-1^int^, and VCAM-1^hi^ subsets in the sdLN and mLN of young and aged mice. **(E)**. Frequency of VCAM-1^lo^, VCAM-1^int^, and VCAM-1^hi^ FRC subsets within young and aged sdLN and mLN stroma. Relative fluorescence intensity (RFI) of surface markers **(F)**. LTBR and **(G)**. SCA-1 on VCAM-1^lo^, VCAM-1^int^, and VCAM-1^hi^ FRC subsets within young and aged sdLN and mLN stroma. RFI normalized to expression on young VCAM-1^hi^ FRCs. Data is representative of lymph nodes retrieved from 6 young and 6 aged mice. Analyzed by two-way ANOVA with adjustment for multiple comparisons, *p*-values: ns, not significant; * < 0.05, ** < 0.01, *** < 0.001, **** < 0.0001.

We used quantification *via* flow cytometry to determine whether aging perturbs the composition of stromal subsets. CD45-depleted cells from enzymatically digested lymph nodes had been separated into skin-draining (sdLN) and mesenteric (mLN) groups, as site-specific differences have been previously reported for young adult mice (Fletcher et al., 2011). FRC, LEC, blood endothelial cell (BEC), and double-negative (DN) subsets were resolved based on PDPN/gp38 and CD31 gating (Figure 5A). We found no significant difference in the frequency of all stromal subsets between young and aged sdLNs (Figure 5B, left panel). This was further shown in the similar numbers of stromal subsets between young and aged sdLNs (Figure 5C, left panel), which reflected the similar overall cellularity of the total lymph node that we observed at this age range (Supplementary Figure S2A). However, we found that FRCs were significantly decreased in frequency within aged mLNs, which was compensated by increased proportions of BECs and DNs (Figure 5B, right panel). This corresponded to a decrease in the FRC numbers in the aged mLNs, though the increase in BEC and DN proportion did not result in a statistically significant increase in their numbers (Figure 5C, right panel). We also observed a trend towards decreased LECs in the aged mLN, though these differences did not rise to the level of statistical significance (Figures 5B,C).

Heterogeneity has been noted within the FRC compartment (Rodda et al., 2018; Pradhan et al., 2021), and MAdCAM-1 expression delineates marginal zone reticular cells (MRCs), which are found in the subcapsular zone and mainly destined to become follicular dendritic cells (Jarjour et al., 2014); we therefore gated out MAdCAM-1^+^ cells from our analysis of FRCs that modulate naïve T cell activity (Supplementary Figure S2B). We subsequently used VCAM-1 upregulation to distinguish stages of FRC differentiation (Bénézech et al., 2010; Fletcher et al., 2015) in the FRCs from young and aged lymph nodes (Figure 5D). We found significantly decreased proportions of VCAM-1^lo^ FRCs in aged sdLNs, with a concomitant increase in aged VCAM-1^int^ FRC frequency (Figure 5E, top panel). Though the overall proportion of FRC from mLN stroma was impacted by aging (Figure 5B, right panel), there was no difference in the distribution of mLN FRC subsets with age (Figure 5E, bottom panel). Thus, we determined that aging only impacted FRC composition at certain lymph node sites, which include the mesenteries. For the skin-draining sites, there was no overall change in FRC proportion, but differences were observed within the compartment when subsetted by VCAM-1 expression.

Flow cytometric analysis further suggested that not only are aged FRC subsets quantitatively different with age, but qualitatively different. We measured surface expression of LTBR, which is highly expressed on FRCs and their progenitors in order to enforce their maturation (Bénézech et al., 2010; Fletcher et al., 2015). In young VCAM-1^lo^ FRCs, LTBR was most highly expressed, and gradually decreased during VCAM-1 upregulation in the sdLN (Figure 5F, top panel). Interestingly, while LTBR expression was similar in young and aged VCAM-1^hi^ FRCs, it was maintained at the same expression level throughout VCAM-1 upregulation (Figure 5F, top panel), indicating that aged FRC progenitors did not have a period of upregulated LTBR expression. Interestingly, the trajectory of LTBR expression was different in the mLNs; in young FRCs, LTBR expression remained constant throughout VCAM-1 upregulation (Figure 5F, bottom panel). LTBR expression was also fairly constant through the aged FRC stages but was about half the magnitude of that in young FRCs (Figure 5F, bottom panel). We further analyzed surface expression of the cell renewal marker SCA-1 (Ly6a), which is also associated with mesenchymal stem cells (Morikawa et al., 2009). In both the sdLN and mLN, SCA-1 was highly expressed in VCAM-1^lo^ FRCs and gradually decreased with VCAM-1 upregulation (Figure 5G). However, the surface expression of SCA-1 was significantly lower in aged VCAM-1^lo^ FRCs at both sites and remained at low levels throughout VCAM-1 upregulation (Figure 5G). Thus, we have detected differences in the frequency of and surface expression of characteristic markers such as LTBR and SCA-1 in VCAM-1^lo^ FRCs, the precursors to mature T cell zone FRCs.

## Discussion

The proportion of naïve T cells is greatly reduced with age, even for lab mice raised in pathogen-free conditions (Thompson et al., 2019), which suggests that the expansion of memory T cells in response to infections cannot be the sole reason for their decline. Changes to the gross structure of lymph nodes have also been described with age, but the mechanisms driving these changes, and whether they influence the maintenance of naïve T cells, has been unclear. 2-photon microscopy has become a standard technique for visualizing the migration of lymphocytes within live tissues (Bajénoff et al., 2006; Lancaster et al., 2019); here we show, for the first time, the application of 2-photon microscopy to imaging within the aged lymph node microenvironment. We found that young, adoptively transferred naïve T cells had decreased mean cell velocities and traveled in increasingly confined trajectories in the aged microenvironment. The aged lymph node had increased infiltration of fibrosis, which encroached on the tissue parenchymal space and severely limited T cell motility in areas of fibrosis. Others have also observed increased fibrosis, in mice and non-human primates (Thompson et al., 2019), as well as in humans (Hadamitzky et al., 2010), and the increase in fibrotic tissue may serve as a physical impediment to T cell motility. Given that within the aged organism, the pool of naïve T cells has reduced infusion of numbers from thymic output, this impairment to T cell motility within the lymph nodes could delay T cell survival crosstalk, escalating the rate of naïve T cell decline.

While our 2-photon imaging indicated that naïve T cells could still home to and migrate within regions within the lymph node to some degree, the age-associated changes to the structure and organization of the lymph node may yet have repercussions; like others (Becklund et al., 2016; Quinn et al., 2018), we observed reduced naïve T cell homeostatic maintenance when young cells were adoptively transferred into sub-lethally irradiated aged hosts. We also observed reduced enrichment of adoptively transferred naïve T cells in aged versus young hosts, suggesting that the aged lymphoid environment could be impaired in recruiting T cells from the circulation, as others have shown in the context of aged infection (Richner et al., 2015). Besides FRCs, LECs also express homeostatic chemokines, and so their involvement in a possible homing defect with age should not be discounted. Thus, in the aged environment, reduced homeostatic turnover and recruitment to the lymph nodes may both contribute to impaired naïve T cell maintenance. On the other hand, *in vitro* co-culture of young naïve T cells with aged stroma was comparable to that of young stroma. The *in vitro* result suggests that aged stroma still retain some ability to promote naïve T cell survival, at least in our short-term experiment. These data suggest that reduced T cell maintenance may not be solely driven by the age of lymph node stroma cells, but rather a consequence of increased fibrosis; the mechanical barrier presented by fibrosis could perhaps be modulated with systemic anti-fibrotic therapies, as has been shown in Pirfenidone treatment of fibrotic lymph nodes induced by human and simian immunodeficiency virus infection (Schaefer et al., 2011; Zeng et al., 2011; Estes et al., 2015).

Paracortical regions in the aged lymph node were characterized by increased expression of αSMA, which was not obvious from the SHG signal observed by 2-photon microscopy. Fibroblasts in lymph nodes and other organs can adopt a fibrotic signature upon administering inflammatory cues (Hou et al., 2018; Li et al., 2020), and as aging is associated with chronic low-level inflammation, it may be driving the increased αSMA signal observed in the aged lymph nodes. The increased lymph node αSMA signal also bore resemblance to the lymph node phenotype of FRC-specific *Ltbr*-deficient mice (*Ccl19*-Cre; *Ltbr* ^fl/fl^) (Chai et al., 2013; Choi et al., 2020). In these young adult mice, the lymph nodes are smaller than WT controls and have decreased T cell cellularity; FRC proportions are also reduced, and reporter-positive FRCs co-express fibrotic markers including αSMA (Chai et al., 2013). FRCs in *Ccl19*-Cre; *Ltbr* ^fl/fl^ mice had reduced levels of ICAM-1 and VCAM-1, suggesting impaired maturation (Chai et al., 2013). Furthermore, FRC expression of CCL21 was abolished, and mice infected with mouse hepatitis virus, normally cleared in WT mice, had diminished immune activation (Chai et al., 2013). Because of these parallels, we measured expression of *Ltbr* in aged stroma, and found it to be decreased. However, expression of LT ligands remained abundant in aged hematopoietic cells. The role of LTβ signaling in early lymph node stromal development is well-appreciated (Bénézech et al., 2010; Brendolan and Caamaño 2012), and its importance in postnatal stromal maintenance has been recently been highlighted (Chai et al., 2013; Li et al., 2019; Shou et al., 2021). However, unlike models of genetically abrogated *Ltbr*, we find a more subtle decrease in stromal *Ltbr* expression with age, and changes to LTBR expression were more plainly revealed once FRCs were subsetted based on their upregulation of VCAM-1. The difference in approach may be why others had concluded that there was no age-associated deficit in LTBR expression in FRCs (Masters et al., 2019). In particular, LTBR expression was most reduced in VCAM-1^lo^ FRCs, which correspond to the most immature FRC subset (Bénézech et al., 2010). Aged VCAM-1^lo^ FRCs also had reduced expression of SCA-1, which suggests they may have lost some of their differentiation potential. Thus, differences in surface LTBR expression suggest FRC subset engagement with LTβ signaling may also differ with age.

Some studies had concluded that FRC composition was reduced with age (Becklund et al., 2016) while others determined that FRC frequency within the stroma was unchanged (Masters et al., 2019) or increased (Turner and Mabbott 2017). While these differences could possibly be explained by differences in enzymatic disassociation and technical approach, we addressed this controversy by analyzing stroma from the skin-draining and mesenteric sites separately, as, like the spleen (Cheng et al., 2019), the stroma of the mesenteries have their own particular developmental trajectory apart from skin-draining sites (Li et al., 2019; Prados et al., 2021). Furthermore, because of their close interaction with the gut tissues and associated microbiota (Bosco and Noti 2021), we postulated that aging may shape the mLNs in a different manner from sdLNs. We found that sdLN FRCs were unchanged with regards to their frequency with age, whereas they were drastically decreased in the mLNs. Within the FRC compartment, we found decreased VCAM-1^lo^ and increased VCAM-1^int^ FRCs within sdLNs, yet unchanged proportions of FRC subsets within the mLNs. As the trajectory of LTBR expression differed between FRC subsets of the sdLN and mLN sites, it appears that LTβ signaling may play distinct roles in promoting FRC differentiation, which could explain disparate changes to FRCs from these different sites with age.

We also measured transcript expression of CCR7 ligands, *Ccl19* and *Ccl21*, responsible for naïve T cell homing and interactions with stroma. We found a trend towards decreased *Ccl19*, though not statistically significant. Changes to *Ccl19* expression may be context-specific, as Becklund and colleagues found reduced expression in peripheral lymph nodes (Becklund et al., 2016). Unlike the former study, we found a significant decrease in *Ccl21* expression by aged stroma, though it is unclear whether they isolated CD45^−^ stroma cells in their analysis, as we had. Expression of *Ccl21* by FRCs transiently decreases in the course of inflammatory activation (Mukherjee et al., 2017). As *Ccl21* expression is modulated by alternative NFκB activation, downstream of LTBR (Brendolan and Caamaño 2012; Mukherjee et al., 2017), its reduction in aged stroma may be driven by the age-associated changes we observed in *Ltbr*.

It remains to be fully explained how aging could lead to impaired LTβ signaling, and whether these changes to the aged lymph node microenvironment directly contribute to naïve T cell decline. Like others, we found that expression of *Il7* was maintained in the aged lymph node stroma (Becklund et al., 2016; Thompson et al., 2019). Becklund and colleagues had suggested that reduced bioavailability of IL-7 drove impaired homeostatic turnover in the aged microenvironment (Becklund et al., 2016); however, unlike Becklund, we did not observe differences in IL-7 signaling in our adoptive transfer experiments. Moreover, unlike earlier experiments by Link and colleagues, we did not observe loss of naïve T cell survival *in vitro* when co-cultured with stroma and IL-7-blocking antibody (Link et al., 2007). Rather, addition of exogenous IL-7 greatly increased T cell numbers when cultured with stroma, and this increase above baseline survival could be blocked by co-incubation with α-IL-7. Interestingly, it has been recently shown with cell-specific knockouts that *Il7* expression by peripheral lymph node stroma was not a requirement for naïve T cell survival (Knop et al., 2020). Thus, IL-7-dependent and -independent mechanisms by which stroma modulate naïve T cell survival are still incomplete. In conclusion, we have characterized the aged lymph nodes as a microenvironment in which fibrosis interferes with T cell motility. Reduction of the CCL21 gradient may further reduce naïve T cell crosstalk with lymph node stroma. Furthermore, impaired expression of LTBR in aged FRCs leads to changes in early stage FRCs. In continuing work, we further investigate the mechanisms by which LTβ signaling is impaired with age, and the consequences that age-associated changes, like fibrosis, have on naïve T cell maintenance and function.

## Data Availability

The original contributions presented in the study are included in the article/Supplementary Material, further inquiries can be directed to the corresponding author.
