# Supplementary material for: Age-Associated Changes to Lymph Node Fibroblastic Reticular Cells
Source: Front Aging. 2022 Jan 25;3:838943. doi: 10.3389/fragi.2022.838943 (PMC9261404; doi:10.3389/fragi.2022.838943)
Supplement: Supplementary file 2 [file DataSheet1.PDF]

SUPPLEMENTARY INFORMATION

Age-associated changes to lymph node fibroblastic reticular cells

T. Kwok, S. C. Medovich, I. Alves da Silva Junior, E. M. Brown, J. C. Haug, M. Barrios, K. A. Morris, and J. N. Lancaster

SUPPLEMENTAL FIGURE 1

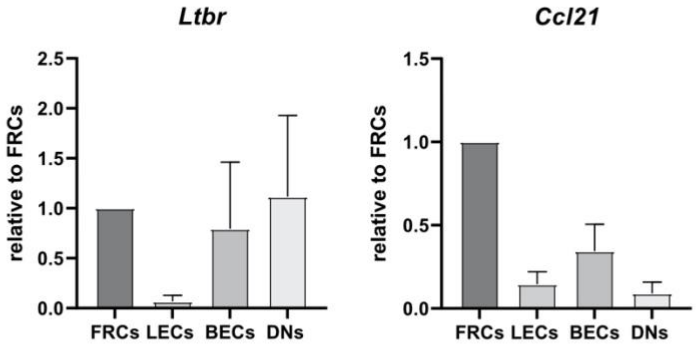

Supplemental Figure 1, related to Figure 4. Stromal subset specific expression of *Ltbr* and *Ccl21*

Lymph nodes from young WT mice were depleted of $CD45^{+}$ cells and sorted into stromal cell subsets before qPCR measurement of *Ltbr* and *Ccl21* expression. Data were normalized to *Actb* expression for each mouse, then normalized to transcript expression by FRCs for the given experiment. Compiled data from 3 experiments, in which lymph node stroma from 3 mice was pooled for each experiment.

SUPPLEMENTAL FIGURE 2

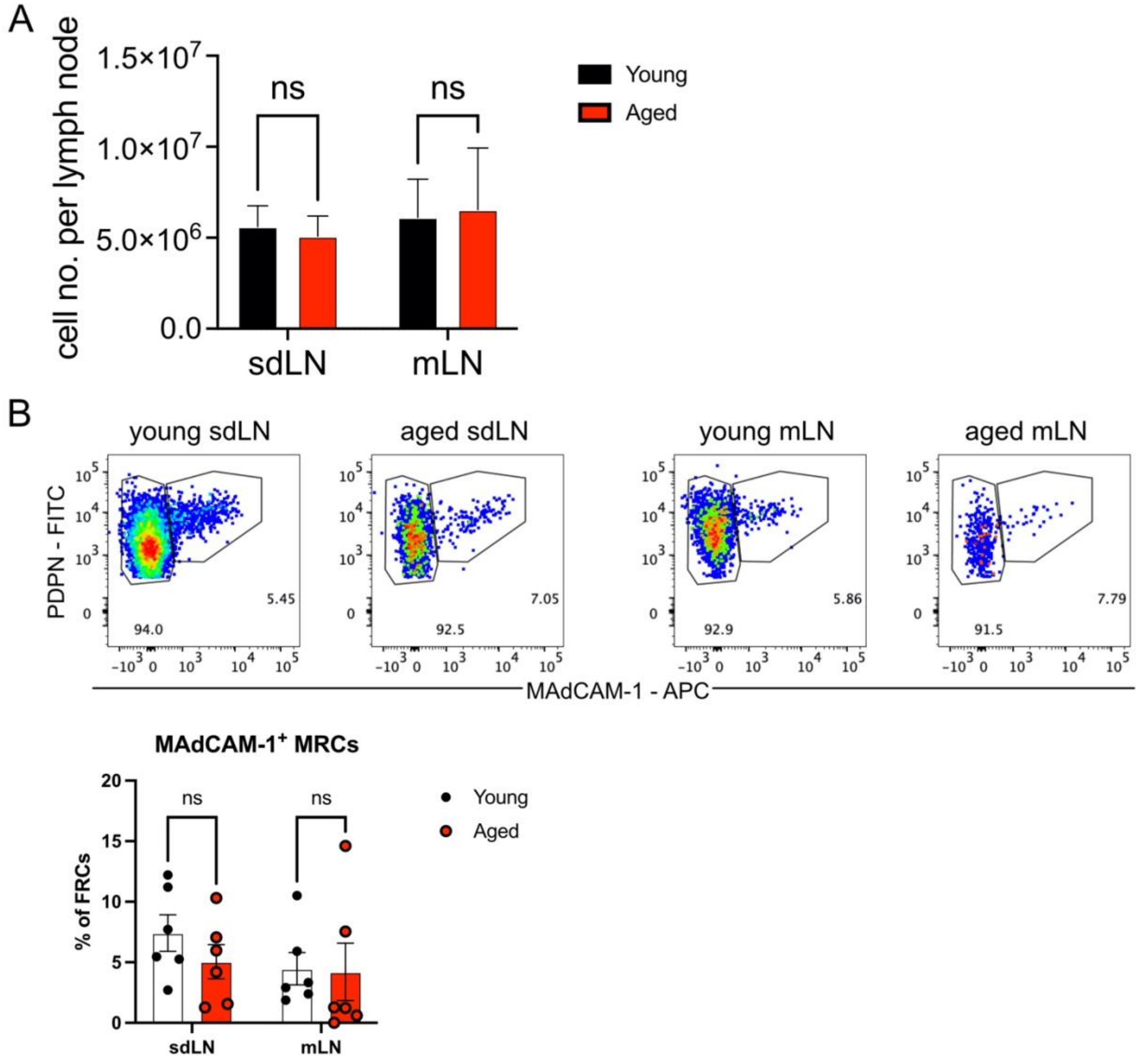

Supplemental Figure 2, related to Figure 5. Comparison of stromal cell composition with age A. Total lymph node cellularity from skin-draining (sdLN) and mesenteric (mLN) lymph nodes harvested from young and aged mice and disassociated by enzymatic digest. **B.** Gating of CD45$^-$ CD35$^-$B220$^-$PDPN$^+$CD31$^-$ FRCs to distinguish MAdCAM-1$^+$ MRCs in the sdLNs and mLNs of young and aged mice (top panel). Frequency of MAdCAM-1$^+$ MRCs of total FRCs in the sdLNs and mLNs of young and aged mice (bottom panel). Compiled data from 6 young and 6 aged mice, analyzed by *t* tests: ns, not significant.

SUPPLEMENTAL MOVIE LEGENDS

Supplementary Movie 1, related to Figure 1A. Migration of $CD8^+$ naïve T cells on a young lymph node slice. GFP^+ $CD8^+$ naïve T cells (green) migrate rapidly within open zones on the lymph node that correspond to the paracortex. Lymph node capsule is defined by second harmonic generation (SHG) of collagen (cyan). Images were acquired for 15 min with 15 sec time intervals, through a depth of 40 μm, and a maximum intensity projection is displayed. Scale bar is 100 μm.

Supplementary Movie 2, related to Figure 1B. Migration of $CD8^+$ naïve T cells on an aged lymph node slice with high fibrosis. GFP^+ $CD8^+$ naïve T cells (green) migrate slowly and in a confined manner within meshwork within zones of the lymph node slice that correspond to the paracortex. Lymph node capsule and meshwork are defined by second harmonic generation (SHG) of collagen (cyan). Images were acquired for 15 min with 15 sec time intervals, through a depth of 40 μm, and a maximum intensity projection is displayed. Scale bar is 100 μm.

Supplementary Movie 3, related to Figure 1B. Migration of $CD8^+$ naïve T cells on an aged lymph node slice with less fibrosis. GFP^+ $CD8^+$ naïve T cells (green) migrate rapidly and subcapsular regions of the aged lymph node with less fibrosis. Lymph node capsule is defined by second harmonic generation (SHG) of collagen (cyan). Images were acquired for 15 min with 15 sec time intervals, through a depth of 40 μm, and a maximum intensity projection is displayed. Scale bar is 100 μm.
